# Comparison of uncalibrated arterial waveform analysis in cardiac surgery patients with thermodilution cardiac output measurements

**DOI:** 10.1186/cc5103

**Published:** 2006-11-21

**Authors:** Michael Sander, Claudia D Spies, Herko Grubitzsch, Achim Foer, Marcus Müller, Christian von Heymann

**Affiliations:** 1 Department of Anesthesiology and Intensive Care Medicine, Charité University Medicine Berlin, Charité Campus Mitte, Campus Virchow Klinikum, Charitéplatz 1, 10117 Berlin, Germany; 2 Department of Cardiovascular Surgery, Charité University Medicine Berlin, Campus Charité Mitte, Charitéplatz 1, 10117 Berlin, Germany

## Abstract

**Introduction:**

Cardiac output (CO) monitoring is indicated only in selected patients. In cardiac surgical patients, perioperative haemodynamic management is often guided by CO measurement by pulmonary artery catheterisation (CO_PAC_). Alternative strategies of CO determination have become increasingly accepted in clinical practice because the benefit of guiding therapy by data derived from the PAC remains to be proven and less invasive alternatives are available. Recently, a device offering uncalibrated CO measurement by arterial waveform analysis (CO_Wave_) was introduced. As far as this approach is concerned, however, the validity of the CO measurements obtained is utterly unclear. Therefore, the aim of this study was to compare the bias and the limits of agreement (LOAs) (two standard deviations) of CO_Wave _at four specified time points prior, during, and after coronary artery bypass graft (CABG) surgery with a simultaneous measurement of the gold standard CO_PAC _and aortic transpulmonary thermodilution CO (CO_Transpulm_).

**Methods:**

Data from 30 patients were analysed during this prospective study. CO_PAC_, CO_Transpulm_, and CO_Wave _were determined in all patients at four different time points prior, during, and after CABG surgery. The CO_PAC _and the CO_Transpulm _were measured by triple injection of 10 ml of iced isotone sodium chloride solution into the central venous line of the PAC. Measurements of CO_Wave _were simultaneously taken at these time points.

**Results:**

The overall correlation showed a Spearman correlation coefficient between CO_PAC _and CO_Wave _of 0.53 (*p *< 0.01) and 0.84 (*p *< 0.01) for CO_PAC _and CO_Transpulm_. Bland-Altman analysis showed a mean bias and LOAs of 0.6 litres per minute and -2.2 to +3.4 litres per minute for CO_PAC _versus CO_Wave _and -0.1 litres per minute and -1.8 to +1.6 litres per minute for CO_PAC _versus CO_Transpulm_.

**Conclusion:**

Arterial waveform analysis with an uncalibrated algorithm CO_Wave _underestimated CO_PAC _to a clinically relevant extent. The wide range of LOAs requires further evaluation. Better results might be achieved with an improved new algorithm. In contrast to this, we observed a better correlation of thermodilution CO_Transpulm _and thermodilution CO_PAC _measurements prior, during, and after CABG surgery.

## Introduction

Advanced haemodynamic monitoring is indicated only in selected patients. In cardiac surgical patients, perioperative haemodynamic management is often guided by cardiac output (CO) measurement using the pulmonary artery catheter (PAC). The use of the PAC, however, has been decreasing over the last years in surgical and cardiac surgical patients as the benefit of guiding therapy is doubtful. Furthermore, its usage might even be associated with increased morbidity [1]. Other randomised studies did not provide clear evidence of benefit or harm by managing critically ill patients with a PAC [2,3]. Only some studies showed beneficial effect by guiding the therapy by PAC-derived data [4]. Therefore, alternative strategies have been developed to measure CO. Aortic transpulmonary thermodilution (CO_Transpulm_), a less invasive technique for determination of the CO, has become increasingly accepted in clinical practice [5-7]. Several investigators established a good correlation between these two methods of CO determination [5-8]. Most devices using transpulmonal thermodilution for CO determination also offer continuous CO determination by arterial pulse contour analysis. In these devices, the initial thermodilution measurement is used to calibrate the algorithm for the continuous CO measurement. Several methodological improvements of the algorithm [9,10] constituted the monitoring of the CO by calibrated continuous arterial pulse contour analysis as an alternative to PAC thermodilution CO (CO_PAC_) in cardiac surgical patients [5,11], showing an accuracy comparable to that of pulmonary artery thermodilution [6,11,12].

Recently, a device offering uncalibrated CO measurement by arterial waveform analysis (CO_Wave_) (Vigileo; Edwards Lifesciences LLC, Irvine, CA, USA) was introduced. As far as this approach is concerned, however, the validity of the CO measurements obtained is utterly unclear. The software of this device calculates CO every 20 seconds on the basis of the last 20-second interval of arterial waveform analysis. The calibration coefficient adjusting for individual characteristics of the vascular resistance and the arterial compliance is re-calculated every 10 minutes on the basis of demographic data and the arterial waveform analysis.

Therefore, the aim of this study was to compare the bias and the limits of agreement (LOAs) (two standard deviations [SDs]) of CO_Wave _at four specified time points prior, during, and after coronary artery bypass graft (CABG) surgery with a simultaneous gold standard thermodilution measurement of CO_PAC _and the thermodilution measurement of CO_Transpulm_.

## Materials and methods

### Patients

After ethical committee approval and written informed consent, 30 patients were considered eligible for this clinical trial from January to April 2006. Inclusion criteria were age more than 18 years and less than 80 years and elective CABG surgery. Exclusion criteria were withdrawal of consent, valve pathologies, left ventricular ejection fraction less than 40%, and symptomatic peripheral artery disease.

### Perioperative management

Oral premedication was with midazolam 0.1 mg/kg. A radial artery was placed in all patients prior to induction of anaesthesia. After induction, a femoral artery was cannulated with a 4-French cannula (Pulsiocath; Pulsion Medical Systems AG, Munich, Germany). A central venous catheter and a PAC (thermodilution catheter; Arrow International, Inc., Reading, PA, USA) were inserted via the right internal jugular vein.

General anaesthesia was induced with etomidate 0.2 mg/kg, fentanyl 5 μg/kg, and pancuronium 0.1 mg/kg. Maintenance was with infusion of fentanyl 5 to 10 μg/kg per hour, boluses of midazolam 0.1 mg/kg, pancuronium 0.03 mg/kg, and 0.6% to 1% end-tidal isoflurane. All patients were ventilated with an oxygen-air mixture (FiO_2 _[inspiratory oxygen fraction] 0.5) to maintain an end-tidal pCO_2 _(partial pressure of carbon dioxide) of 35 to 45 mm Hg. Cardiopulmonary bypass (CPB) technique was normothermic using intermittent antegrade warm blood cardioplegia as described by Calafiore and colleagues [13]. Transfusion management was performed according to our standard operating procedure [14]. Durations of anaesthesia, surgery, and aortic occlusion and number of CABGs were recorded.

### Determination of CO

CO was determined at four time points. The first measurement was performed after induction of anaesthesia and placement of the catheters. The second measurement was performed 15 minutes after sternotomy prior to CPB. The third and fourth measurements were performed one hour after admission to the intensive care unit (ICU) and six hours after admission to the ICU, respectively. A stable haemodynamic condition was a prerequisite for the measurements. Therefore, infusion of large volumes of colloids or cristalloids or the bolus administration of vasopressors was not permitted during the measurements. The CO_PAC _and the CO_Transpulm _were measured by triple injection of 10 ml of iced isotone sodium chloride solution into the central venous line of the PAC. The CO_PAC _and the CO_Transpulm _were calculated by commercially available monitors (CCO module, Solar 8000; Marquette Hellige GmbH, Freiburg, Germany, and PiCCO CCO monitor; Pulsion Medical Systems AG, München, Germany). In case of a deviation of more than 10% of a measurement, five measurements were performed and the highest and lowest were rejected. The CO_PAC _and the CO_Transpulm _measurements were carried out simultaneously.

The measurement of CO_Wave _was performed by arterial waveform analysis without any external calibration by using a commercially available transducer (FloTrac; Edwards Lifesciences LLC), which links the radial arterial line with the monitor (Vigileo; Edwards Lifesciences LLC). A stable haemodynamic condition with no damping of the arterial pressure line, which could be achieved in all patients, was also a prerequisite for this measurement. For each measurement of CO_PAC _and CO_Transpulm_, a corresponding simultaneous CO_Wave _was documented.

### Statistical analysis

All data are expressed as mean and standard error of the mean. Statistical analysis was performed by linear regression analysis. Bias and LOAs (two SDs) were assessed according to the method described by Bland and Altman [15]. The percentage error was calculated according to the method described by Critchley and Critchley [16]. All numerical calculations were carried out with SPSS for Windows, Release 11.5.1 (SPSS Inc., Chicago, IL, USA).

## Results

During this study, we evaluated CO using three different methods. To do so, we performed 120 measurements of CO in 30 patients at four different time points. In one patient, inserting the PAC was impossible. In another patient, we were unable to place the arterial thermodilution catheter. Due to technical problems with the transducer, the uncalibrated arterial waveform CO could not be analysed in six measurements in five patients. In one patient, postoperative measurements were impossible because this patient received an intra-aortic balloon pump for weaning from CPB. As a result, we were able to analyse 110 paired measurements comparing CO_PAC _with CO_Transpulm _and 108 paired measurements comparing CO_PAC _with CO_Wave_.

**Figure 1:**
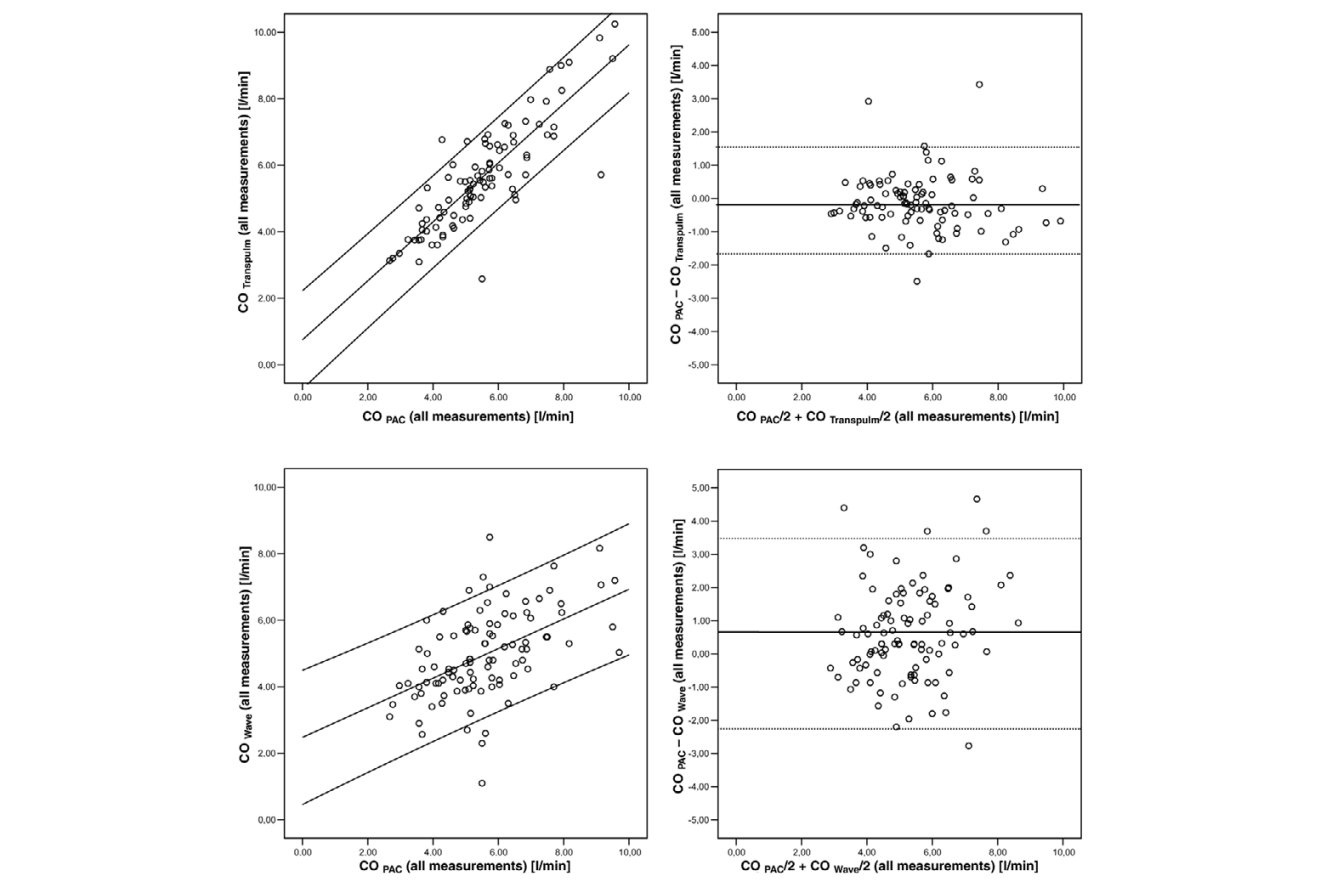
Regression analysis and Bland-Altman plots of CO_PAC _versus CO_Wave _and of CO_PAC _versus CO_Transpulm _for overall measurements. CO_PAC_, pulmonary artery catheter thermodilution cardiac output; CO_Transpulm_, aortic transpulmonary thermodilution cardiac output; CO_Wave_, uncalibrated pulse contour cardiac output.

Anaesthesia and surgery were uncomplicated in all patients. Patients' basic characteristics are given in Table 1. Surgery- and ICU-related data are also provided in Table 1. Haemodynamic data are provided in Table 2. Heart rate increased significantly at all points of measurement compared with baseline values (*p *< 0.01). Only prior to CPB was the central venous pressure significantly decreased compared with the baseline measurement (*p *= 0.04). The overall correlation between CO_PAC _and CO_Wave _was 0.53 (*p *< 0.01) (Figure 1), whereas the overall correlation between CO_PAC _and CO_Transpulm _was 0.84 (*p *< 0.01) (Figure 1). Bland-Altman analysis showed a mean bias and LOAs of 0.6 litres per minute and -2.2 to +3.4 litres per minute for CO_PAC _versus CO_Wave _(Figure 1) and -0.1 litres per minute and -1.8 to +1.6 litres per minute for CO_PAC _versus CO_Transpulm_. The percentage errors for CO_PAC _versus CO_Wave _and for CO_PAC _versus CO_Transpulm _were 54% and 30%, respectively.

**Figure 2:**
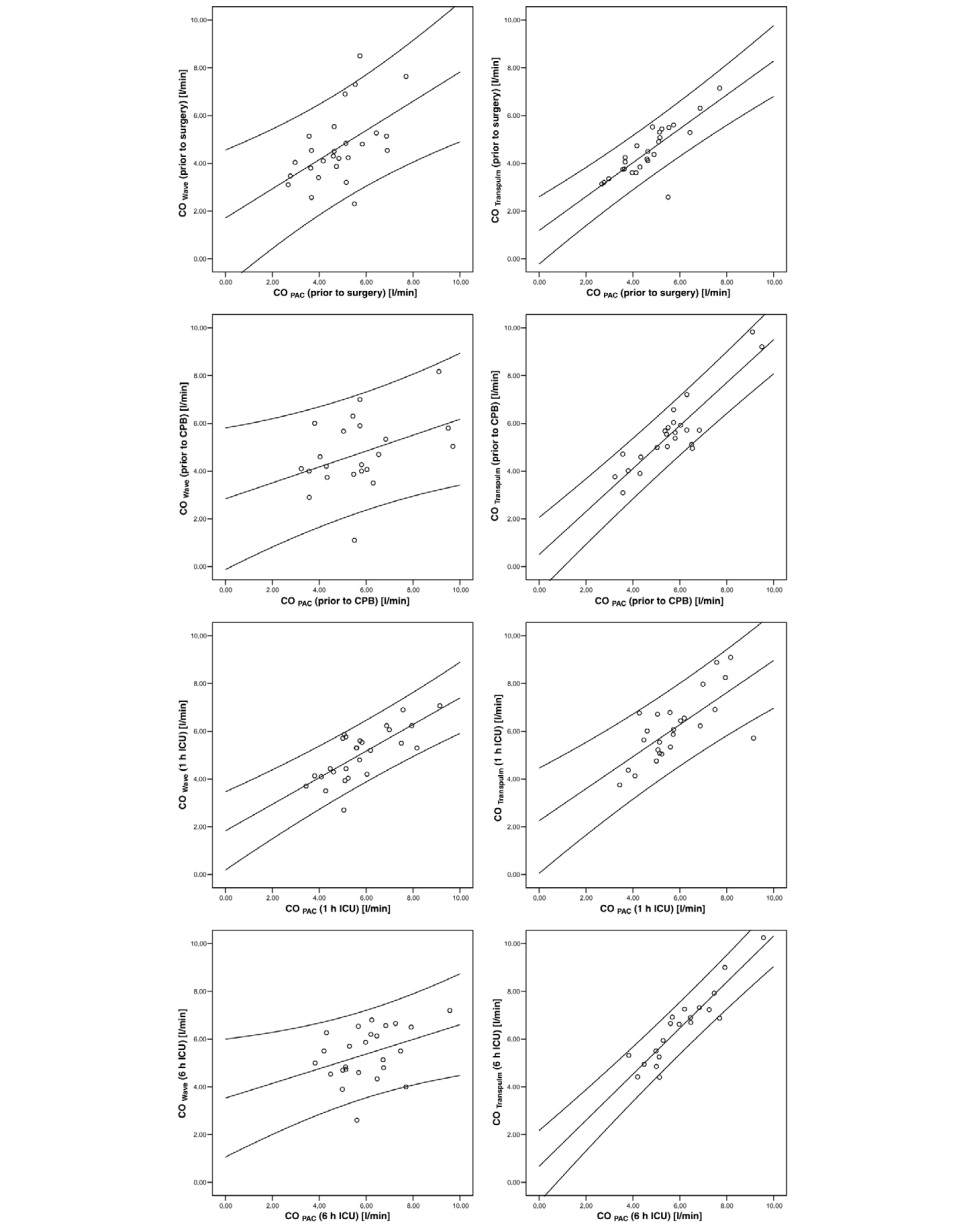
Regression analysis and Bland-Altman plots of CO_PAC _versus CO_Wave _and of CO_PAC _versus CO_Transpulm _for each individual point of measurement. CO_PAC_, pulmonary artery catheter thermodilution cardiac output; CO_Transpulm_, aortic transpulmonary thermodilution cardiac output; CO_Wave_, uncalibrated pulse contour cardiac output; CPB, cardiopulmonary bypass; 1 h ICU, one hour after admission to the intensive care unit; 6 h ICU, six hours after admission to the intensive care unit.

**Figure 3:**
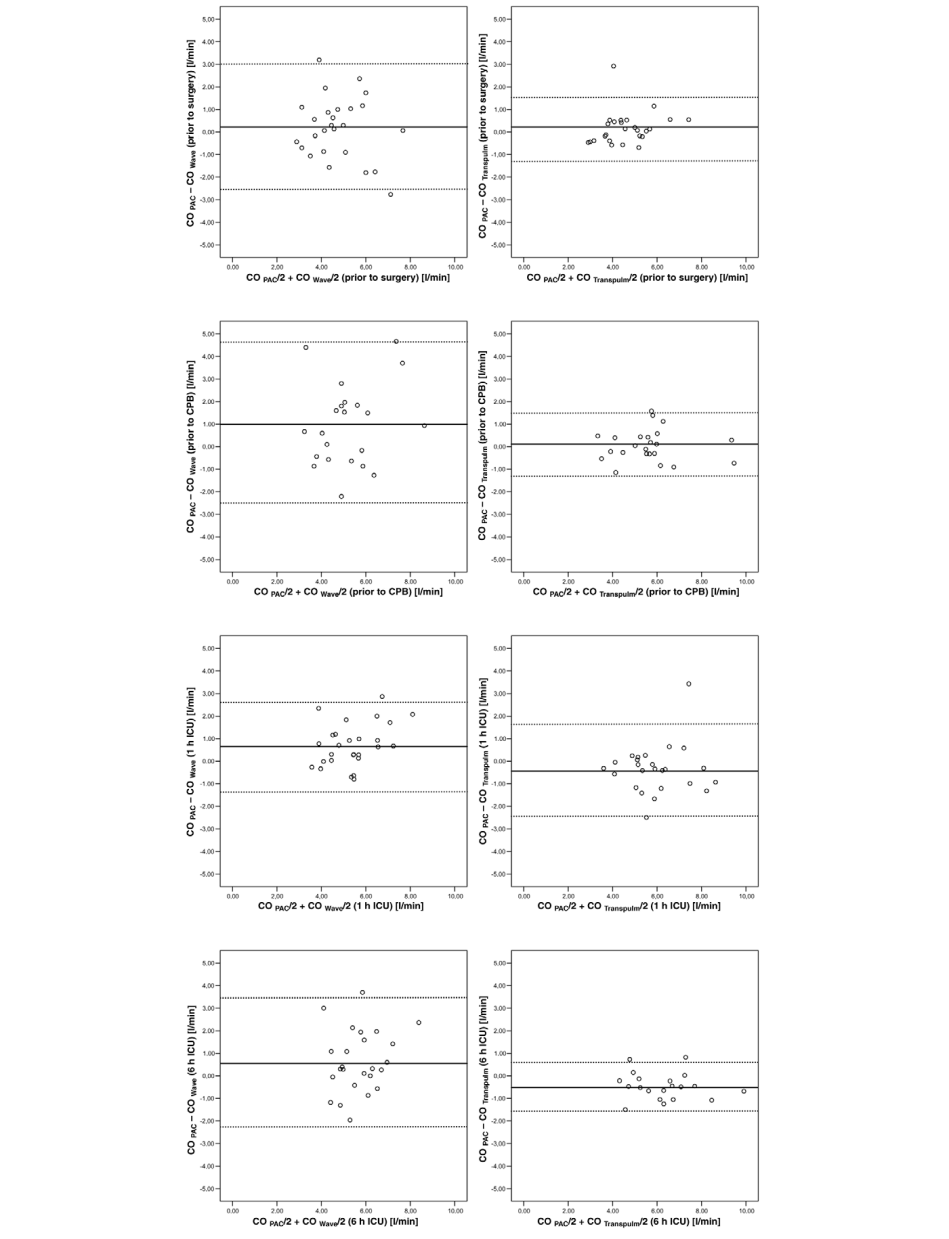
Bland-Altman plots of CO_PAC _versus CO_Wave _and of CO_PAC _versus CO_Transpulm _for each individual point of measurement. CO_PAC_, pulmonary artery catheter thermodilution cardiac output; CO_Transpulm_, aortic transpulmonary thermodilution cardiac output; CO_Wave_, uncalibrated pulse contour cardiac output; CPB, cardiopulmonary bypass; 1 h ICU, one hour after admission to the intensive care unit; 6 h ICU, six hours after admission to the intensive care unit.

Prior to surgery, CO_PAC _and CO_Wave _showed a correlation coefficient of 0.54 (*p *< 0.01) and CO_PAC _and CO_Transpulm _a coefficient of 0.78 (*p *< 0.01) (Figure 2). Bland-Altman analysis for CO_PAC _versus CO_Wave _showed a mean bias and LOAs of 0.2 litres per minute and -2.6 to +3.0 litres per minute and CO_PAC _versus CO_Transpulm _of 0.2 litres per minute and -1.2 to +1.6 litres per minute (Figure 3). The percentage errors for CO_PAC _versus CO_Wave _and for CO_PAC _versus CO_Transpulm _were 58% and 32%, respectively. There was no correlation between CO_PAC _and CO_Wave _(correlation coefficient of 0.29) (Figure 2), whereas the correlation coefficient between CO_PAC _and CO_Transpulm _prior to CPB was 0.74 (*p *< 0.01). At this time point, the Bland-Altman analysis showed a mean bias and LOAs of +1.0 litres per minute and -2.6 to +4.6 litres per minute for CO_PAC _versus CO_Wave _and 0.1 litres per minute and -1.3 to +1.5 litres per minute for CO_PAC _versus CO_Transpulm _(Figure 3). The percentage errors for CO_PAC _versus CO_Wave _and for CO_PAC _versus CO_Transpulm _were 70% and 25%, respectively.

After admission to the ICU, CO_PAC _versus CO_Wave _and CO_PAC _versus CO_Transpulm _showed a reasonable correlation, with correlation coefficients of 0.69 (*p *< 0.01) and 0.68 (*p *< 0.01), respectively (Figure 2). Bland-Altman analysis established a mean bias and LOAs of 0.7 litres per minute and -1.3 to +2.7 litres per minute versus -0.4 litres per minute and -2.6 to +1.8 litres per minute, respectively (Figure 3). The percentage errors for CO_PAC _versus CO_Wave _and for CO_PAC _versus CO_Transpulm _were 36% and 36%, respectively. Six hours after ICU admission, the comparison of CO_PAC _versus CO_Wave _and CO_PAC _versus CO_Transpulm _resulted in correlation coefficients of 0.36 (not significant) and 0.88 (*p *< 0.01), respectively (Figure 2). Bland-Altman analysis showed a mean bias and LOAs of -0.5 litres per minute and -1.7 to +0.7 litres per minute versus 0.6 litres per minute and -2.2 to +3.4 litres per minute, respectively (Figure 3). The percentage errors for CO_PAC _versus CO_Wave _and for CO_PAC _versus CO_Transpulm _were 48% and 19%, respectively.

The change in CO between two subsequent measurements prior to surgery and prior to CPB, prior to CPB and admission to the ICU, and between admission to the ICU and six hours later were, for CO_PAC_, 1.2 (1.5), -0.2 (1.8), and 0.3 (1.4), respectively. The changes for CO_Wave _were 0.4 (2.0), 0.4 (1.4), and 0.2 (1.3), respectively. For the change of CO_Transpulm_, the corresponding values were 1.3 (1.6), 0.4 (1.6), and 0.3 (1.4), respectively. Correlation coefficients of the change in CO_PAC _versus CO_Wave _and CO_PAC _versus CO_Transpulm _between measurements prior to surgery and prior to CPB were 0.55 (*p *< 0.01) and 0.82 (*p *< 0.01), respectively. Between measurements prior to CPB and admission to the ICU, the coefficients were 0.51 (*p *= 0.2) and 0.67 (*p *< 0.01), respectively, and 0.60 (*p *< 0.01) and 0.44 (*p *= 0.05), respectively, for measurements between admission to the ICU and six hours later.

## Discussion

This is the first study evaluating a new method of estimating uncalibrated arterial waveform CO in comparison with two standard methods of CO determination. The most important finding of our study was that intraoperative and early postoperative CO measurements by the uncalibrated arterial waveform analysis showed a high bias and a wide range of LOAs in comparison with the CO_PAC _measurement, which was the reference method in this study. In contrast, we found a better correlation between CO_PAC _and transpulmonal thermodilution CO measurement CO_Transpulm_.

In this study, we evaluated the FloTrac sensor and the Vigileo monitor system for continuous monitoring of CO. This system does not require thermodilution or dye dilution. Rather, it bases its calculations on arterial waveform characteristics in conjunction with patient demographic data. The software for this device calculates CO every 20 seconds on the basis of the last 20-second interval of arterial waveform analysis. The calibration coefficient adjusting for individual characteristics of the vascular resistance and the arterial compliance is re-calculated every 10 minutes on the basis of demographic data and the arterial waveform analysis. In contrast to similar devices analysing the arterial waveform, this device does not require calibration with another method [17] and uses a radial artery only. So far, however, there have not been any controlled peer-reviewed studies comparing this method with standard methods of CO determination.

This trial investigated the validity of continuous CO measurement by uncalibrated arterial waveform analysis compared with standard techniques (CO_PAC _and CO_Transpulm_) prior, during, and after CABG surgery. We could demonstrate that all techniques of CO measurement have their technical limitations, including difficulties with correct catheter placement, transducer malfunction, and CO monitor malfunction. In our intraoperative and early postoperative setting in patients undergoing cardiac surgery, we found the use of the PAC with fast determination of the CO by thermodilution and high precision within one set of measurement was the best alternative of CO determination. The main practical advantage of CO_Wave _measurement in this setting is that it is a quick and easy way of determining CO. The algorithm of the CO monitor automatically starts to determine the CO by continuous arterial waveform analysis in all patients with pulsatile flow. Therefore, in the setting of CABG surgery, haemodynamic monitoring using a pulse contour device with a fast and continuous approach might be practical and advantageous for haemodynamic-oriented therapy. The anaesthetist can direct his/her full attention on vasoactive and volume therapy, which might sometimes be necessary in unstable CABG patients in the perioperative period, rather than be involved in cumbersome, time-consuming, intermitted thermodilution techniques of CO determination. These advantages are, however, only relevant if the data obtained are valid.

Overall analysis of all CO_Wave _measurements pooled failed to show a clinically acceptable correlation and LOAs in comparison with the total of CO_PAC _measurements. We were unable to show a reliable correlation between CO_PAC _and CO_Wave _prior to CPB and six hours after admission to the ICU. The best correlation was observed one hour after admission to the ICU, with a correlation coefficient of 0.68. Even at this time point, however, the bias and the LOAs were unacceptably high (0.7 litres per minute and -1.3 to +2.7 litres per minute). This was, however, the only time point when the bias and the LOAs between CO_PAC _and CO_Transpulm _were also unacceptably high (-0.4 litres per minute and -2.6 to +1.8 litres per minute). All other measurements between CO_PAC _and CO_Transpulm _showed clinically acceptable bias and LOAs. As far as we know, there are no other controlled studies investigating uncalibrated arterial waveform analysis in comparison with standard methods of CO determination.

Pulse contour analysis CO has been established as a valid and cost-effective device for CO determination after calibration [18,19]. Most devices providing continuous pulse contour analysis, however, need calibration by an independent method of CO measurement. After calibration by either thermodilution or lithium dilution CO measurement, pulse contour CO algorithms displayed a clinically acceptable bias and LOAs [6,18,20].

Previous investigations with calibrated pulse contour analysis showed only a reasonable correlation with thermodilution methods of CO determination, with a bias and LOAs of -0.2 litres per minute and -2.2 to +2.6 litres per minute after cardiac surgery [6]. Therefore, we suggest that CO determination with pulse contour analysis in a setting after cardiac surgery might not be the ideal method [21]. Uncalibrated arterial waveform analysis in this setting might even yield worse results. This conclusion is in line with our findings.

We compared overall calibrated CO_Transpulm _measurement performed by aortic transpulmonary CO determination with overall CO_PAC_. We found a better correlation between the CO_Transpulm _and the CO_PAC _[5,6,22] with the exception of the time point one hour after admission to the ICU. The greater scatter between the two CO measurements after admission to the ICU compared with all other measurements may have been the influx of cooler blood derived from compartments, which might be hypoperfused during and early after CPB and then reperfused during the first hours after surgery as suggested by previous investigators [5,23]. A decrease in body temperature worsens the signal-to-noise ratio of the thermal indicator used for determination of the CO by these methods. In this setting, better results might be achieved by using an indicator independent from thermal signals.

A limitation of our study concept is that we do not know the 'true' CO. Bearing in mind, however, that we did find a rather good correlation for the two thermodilution measurements, we assume that thermodilution-derived CO determination represents a reliable estimation of the 'true' CO in clinical practice. The use of the radial artery for CO_Wave _determination, which was in line with the recommendations of the manufacturer, might have influenced the accuracy of the CO determination due to vasoconstriction. However, because no patient received continuous norepinephrine, we suggest that vasoconstriction might not be the main factor influencing the accuracy of the CO determination with this method.

## Conclusion

Our study of arterial waveform analysis with an uncalibrated algorithm showed that CO_Wave _underestimated CO_PAC _to a clinically relevant extent in the difficult setting prior, during, and early after CABG surgery with the software used in this study. The wide range of LOAs requires further evaluation. In contrast to this, we observed a better correlation of calibrated CO_Transpulm _and CO_PAC _measurements prior, during, and after CABG surgery.

The bias and LOAs of CO_Wave _need to be evaluated in different settings against standard methods of CO measurements to prevent patients from being exposed to wrong therapeutic decisions. However, the new software version of this device, featuring a shorter recalibration period, might lead to better results and has to be re-evaluated in this setting.

## Key messages

• We observed a good correlation of CO_Transpulm _and CO_PAC _measurements prior, during, and after CABG surgery.

• Our study could not establish pulse contour analysis with an uncalibrated pulse contour algorithm to be a method yielding reliable results under difficult conditions in perioperative CABG patients.

• CO_Wave _underestimated CO_PAC _and showed a wide range of LOAs, requiring further clinical evaluation in different patient populations.

## Abbreviations

CABG = coronary artery bypass graft; CO = cardiac output; CO_PAC _= pulmonary artery catheter thermodilution cardiac output; CO_Transpulm _= aortic transpulmonary thermodilution cardiac output; CO_Wave _= uncalibrated pulse contour cardiac output; CPB = cardiopulmonary bypass; ICU = intensive care unit; LOA = limit of agreement; PAC = pulmonary artery catheter; SD = standard deviation.

## Competing interests

This study was financially supported by Edwards Lifesciences LLC.

## Authors' contributions

MS and CvH prepared the manuscript, carried out the cardiac output measurements, conceived the study, and performed the statistical analysis. AF and MM helped with the recruitment of the patients and the drafting of the manuscript. HG participated in the study design and helped with the recruitment of patients. CS drafted the manuscript and helped with the study design and coordination. All authors read and approved the final manuscript.

## Figures and Tables

**Table 1:** Patients' basic characteristics and surgery-related data

	*n*	Mean	SD
Age (years)	30	67	7.6
Gender (male/female)	30	24/6	
Height (cm)	30	173	8.9
Weight (kg)	30	82	9.3
Body mass index (kg/m^2^)	30	27	2.8
Urine volume during CPB (ml)	30	391	185
Urine volume during surgery (ml)	30	1,030	324
Duration of anaesthesia (minutes)	30	312	56
Duration of surgery (minutes)	30	208	52
CPB time (minutes)	30	91	29
Aortic cross-clamp time (minutes)	30	55	23
APACHE II score	30	16	6

APACHE, acute physiology and chronic health evaluation; CPB, cardiopulmonary bypass; SD, standard deviation.

**Table 2:** Haemodynamic data

	*n*	Mean	SD
After induction of anaesthesia			
Heart rate (beats per minute)	30	69	16
MAP (mm Hg)	30	71	15
PMAP (mm Hg)	30	19	5
CVP (mm Hg)	30	9	5
PVR (dyn/s per cm^-5^)	30	184	245
SVR (dyn/s per cm^-5^)	30	1,031	342
CO_PAC_	30	4.79	1.23
CO_Wave_	30	4.66	1.52
CO_Transpulm_	30	4.50	1.07
After sternotomy			
Heart rate (beats per minute)	30	76*	12
MAP (mm Hg)	30	68	13
PMAP (mm Hg)	30	19	5
CVP (mm Hg)	30	7*	4
PVR (dyn/s per cm^-5^)	30	325	492
SVR (dyn/s per cm^-5^)	30	945	338
CO_PAC_	30	5.74	1.73
CO_Wave_	30	4.69	1.44
CO_Transpulm_	30	5.48	1.53
One hour after admission to ICU			
Heart rate (beats per minute)	30	79*	15
MAP (mm Hg)	30	72	13
PMAP (mm Hg)	30	21	6
CVP (mm Hg)	30	9	5
PVR (dyn/s per cm^-5^)	30	225	463
SVR (dyn/s per cm^-5^)	30	938	220
CO_PAC_	30	5.75	1.41
CO_Wave_	30	5.02	1.04
CO_Transpulm_	30	6.01	1.41
Six hours after admission to ICU			
Heart rate (beats per minute)	30	81*	13
MAP (mm Hg)	30	73	10
PMAP (mm Hg)	30	21	7
CVP (mm Hg)	30	9	5
PVR (dyn/s per cm^-5^)	30	128	48
SVR (dyn/s per cm^-5^)	30	914	280
CO_PAC_	30	6.03	1.34
CO_Wave_	30	5.25	1.11
CO_Transpulm_	30	6.33	1.51

*significant change compared to baseline. CO_PAC_, pulmonary artery catheter thermodilution cardiac output; CO_Transpulm_, aortic transpulmonary thermodilution cardiac output; CO_Wave_, uncalibrated pulse contour cardiac output; CVP, central venous pressure; ICU, intensive care unit; MAP, mean arterial pressure; PMAP, peripheral mean arterial pressure; PVR, pulmonary vascular resistance; SD, standard deviation; SVR, systemic vascular resistance.
